# Dataset of single *Mycobacterium tuberculosis* bacteria cells with different antibiotic susceptibility obtained by Raman spectroscopy

**DOI:** 10.1016/j.dib.2018.11.095

**Published:** 2018-11-23

**Authors:** Andrey Zyubin, Anastasia Lavrova, Olga Manicheva, Marine Dogonadze, Ilia Samusev

**Affiliations:** a Immanuel Kant Baltic Federal University, A. Nevskogo St. 14, 236016 Kaliningrad, Russia; b Research Institute of Phthisiopulmonology, Polytechnicheskaya 32, 194064 Saint Petersburg, Russia; c Saint-Petersburg State University, 7/9 Universitetskaya Emb., 199034 Saint Petersburg, Russia

**Keywords:** Raman spectroscopy, *Mycobacterium tuberculosis*, multidrug resistance (MDR) strains, sensitive drug (SD) strains, spectral data

## Abstract

This data article contains Raman experimental data, obtained with Horiba Jobin-Yvon LabRam HR800 spectrometer (Japan), which can be used for rapid identification of *Mycobacterium tuberculosis* (*MbT*) bacteria (Beijing clade) *in vitro*. Data present analyzed Raman spectra of bacterial cells with various drug resistances obtained from pulmonary and extra pulmonary samples. Data can provide information about characteristic maxima of different structures in biological cell.

**Specifications table**

**Table:** 

Subject area	*Physics*
More specific subject area	*Raman spectroscopy of bacterial cells*
Type of data	*Figures*
How data were acquired	*Horiba Jobin-Yvon LabRam HR800 (Jobin-Yvon Ltd., Japan) Raman spectrometer*
Data format	*Analyzed*
Experimental factors	*Bacterial cells isolated from the pulmonary and extra pulmonary samples have been cultured on Lowenstein – Jensen medium at 37 °C*
Experimental features	*Raman spectra were obtained with LabRam HR800 spectrometer (Japan), using 632,8 nm He–Ne laser. Explanation of spectra has been performed using 600–1800 cm*^*−1*^*region and KnowItAll (Biorad) software.*
Data source location	*Kaliningrad, Russian Federation*
Data accessibility	*Data are presented in this article*
Related research article	*A. Zyubin, A. Lavrova, O. Manicheva, M. Dogonadze, A. Tsibulnikova & I. Samusev, Methodology of mycobacteria tuberculosis bacteria detection by Raman spectroscopy, Nanophotonics Australasia 2017. Vol. 10456. International Society for Optics and Photonics, 2018*[1].

**Value of the data**

● Raman spectra can be used for bacterial identification *in vitro*.
● Raman spectra can be used to reveal spectral differences for sensitive (**SD**) and multidrug resistant (**MDR**) strains for pulmonary and extra pulmonary samples.
● Raman data can be used as a supplementary tool in strain antibiotic susceptibility routine analysis.

## 1 Data

**Fig. 1:**
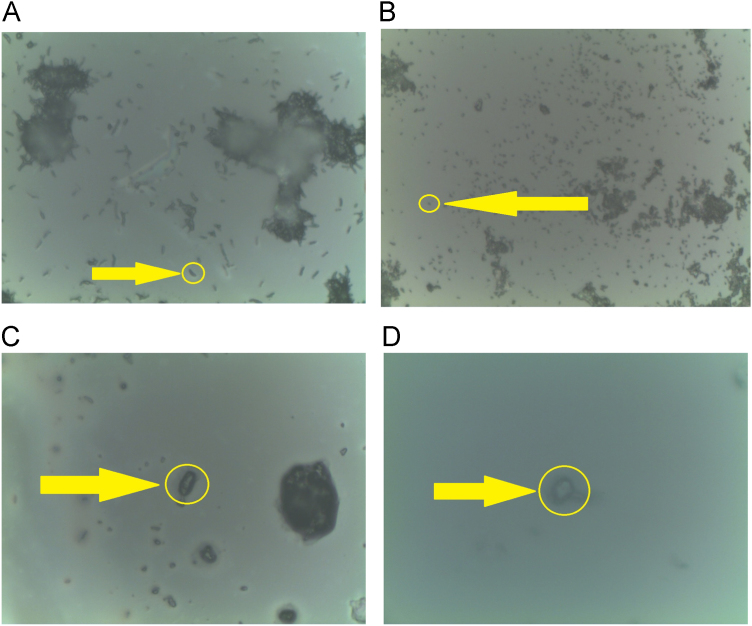
Laser position in inactivated bacterial culture (yellow rounds and arrows mark the laser beam focus on the single cell). a) ×50 optical image of *MbT* pulmonary SD culture; b) x50 optical image of *MbT* extra pulmonary SD culture; c)×100 optical image of *MbT* pulmonary MDR culture; d) ×100 optical image of *MbT* extra pulmonary MDR multi-drug sensitive culture.

**Fig. 2:**
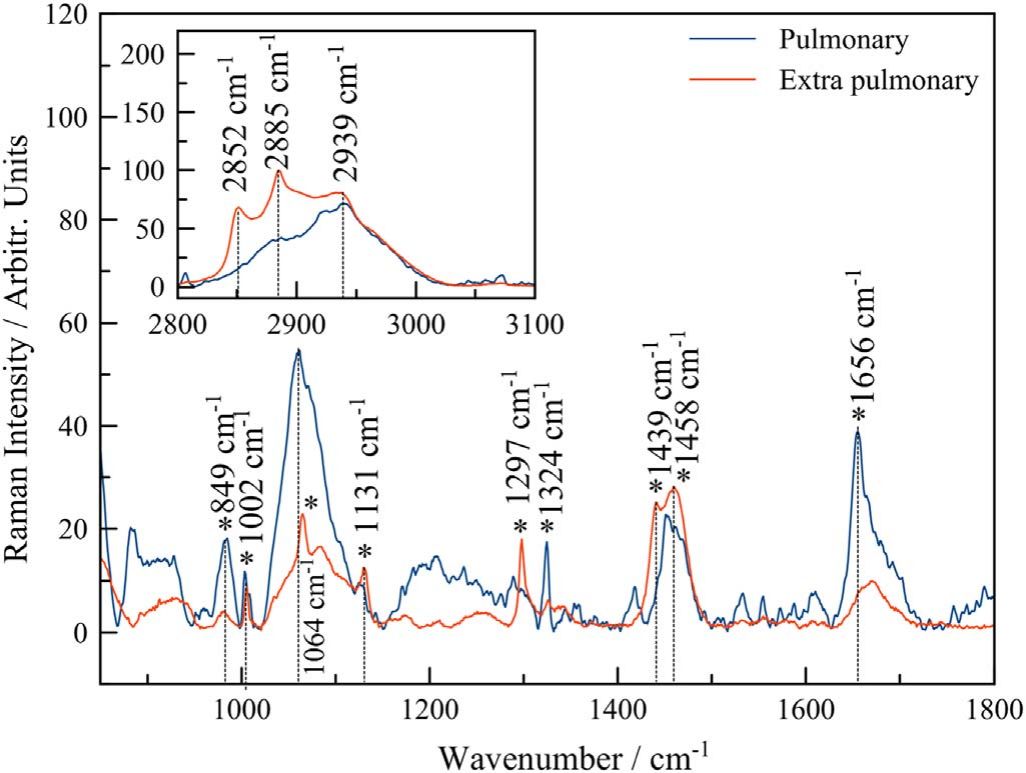
Raman spectra of MbT obtained from pulmonary (blue line) and extra pulmonary (red line) samples. All data are presented for SD strains. High wave number region has been performed at top left corner.

**Fig. 3:**
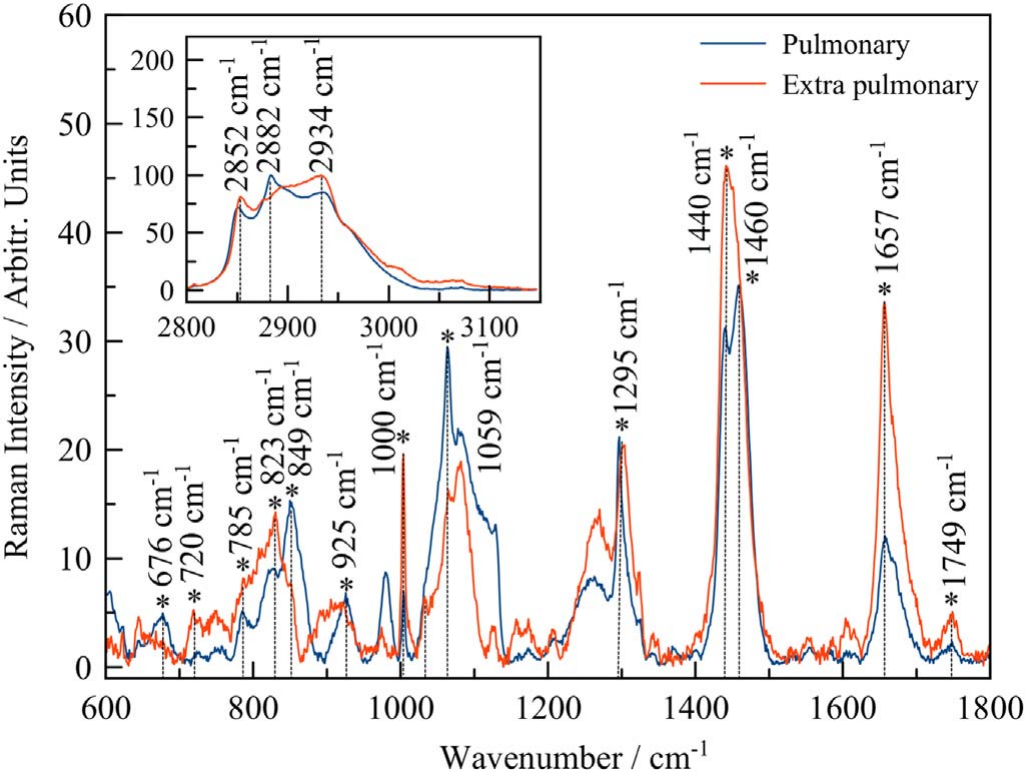
Raman spectra of MbT obtained from pulmonary (blue line) and extra pulmonary (red line) samples. All data are presented for MDR strains. High wave number region has been performed at top left corner.

In this data article, we present data on the Raman spectroscopy for *Mycobacterium tuberculosis* (*MbT*) bacterial cells. The presented data include optical images of bacterial samples (Fig. 1), as well as Raman spectra for sensitive and multidrug resistant bacteria for pulmonary (Fig. 2) and extra pulmonary (Fig. 3) Beijing strains. The main characteristic bands also have been marked. The main vibrational bands positions are presented in Table 1.

**Table 1:** Main vibrational characteristic bands.

**Pulmonary**		**Extra pulmonary**		**Vibrational modes**
**Sensitive**	**Medium drug resistant**	**Sensitive**	**Medium drug resistant**	
849 mbr	849 wbr	847 mbr	823 mbr	Ring-breathing mode of tyrosine with the CaDPA carboxylate stretching mode
1002 m	1002 m	1000 s	1000 m	ν (CC) Aromatic ring breathing (phenylalanine)
1064 s	1064 m	1059 s	1059 vs	C–C,C–N
–	1297 m	1295 s	1295 s	CH_2_ twist vibration (lipids)
1458 s	1458 s	1460 vs	1460 s	CH_2_/CH_3_ deformation
1656 s	1656 s	1657 s	1657 s	Amide I
–	2852 m	2851 m	2852 m	CH_2_
–	2885 m	2882 m	–	ν(CH_2_)_sym_

Abbreviations: w, weak; mw, medium weak; m, medium; ms, medium strong; s, strong; vs, very strong; sh, shoulder; br, broad; δ: deformation vibration, ν: stretching vibration.

## 2 Materials and methods

### 2.1 Sample preparation

Clinical strains have been selected from the collection of St. Petersburg Research Institute of Phthisiopulmonology. Selected strains were isolated from patient specimen: pulmonary (sputum) and extra-pulmonary (surgical bone material). A heavy suspension of the cell mass in a physiological solution with 15% glycerol was stored at −80 °C, and then it was washed by distillate. To inactivate bacterial cells, 250 µl of the defrosted suspension has been placed in 11 ml of distilled water and then heated for 20 minutes at +80 °C. After centrifugation (2000 rpm, 10 min), the pellet was re-suspended in 100 µl of distilled water. The drug sensitivity of clinical strains has been determined by the standard absolute concentrations method on the Lowenstein-Jensen medium and/or by the BACTEC MGIIT 960 using the manufacturer׳s protocol.

### 2.2 Raman experiment

Raman spectra were obtained by Raman LABRAM HR 800 (Jobin-Yvon Ltd., Japan) spectrometer, using the 632.8 nm He-Ne laser excitation with 30 mW power on sample. The optical scheme included Olympus BX 41 microscope with 100×(NA 0.9) and 50×(NA 0.75) objectives. Spectrometer had a focal length of 800 mm with 600 g/mm holographic diffraction grating and was equipped with a CCD camera with 1024×256 pixels. Wavenumber accuracy was 1 cm^−1^. Spectral resolution was 1.5 cm^−1^.

The laser spot size ranged from 1×25 µm to 1×30 µm and was positioned at the bacterial single cell. Rayleigh scattering was eliminated by the notch filters (Fig. 1a-d).

The quartz sample holder was mounted on a standard stage for an Olympus BX41 microscope. 10 µl droplet of bacterial suspension with different antibiotic susceptibility was put on quartz glass, dried for 2 minutes at room temperature, and then placed to the microscope holder. Averaged spectra have been collected from each sample with certain antibiotic susceptibility. The instrument was calibrated with silicon at a static spectrum centered at 520.1 cm^−1^ for 1 s. Sample holder was mounted on a standard stage for an Olympus BX41 microscope. Droplets of bacterial suspension were put on quartz glass, dried for 2 minutes and then placed to the microscope holder (as mentioned above). After registration, the spectra were saved as.txt and specific Horiba format (.ngs) on PC, connected to the Raman unit.

KnowItAll Vibrational Spectroscopy Edition (BioRad, USA) was used for linear baseline correction, Savitsky-Golay smoothing and normalization of all registered spectra and further analysis of peaks position and their intensity.
